# Palladium-Catalyzed Heck Coupling Reaction of Aryl Bromides in Aqueous Media Using Tetrahydropyrimidinium Salts as Carbene Ligands 

**DOI:** 10.3390/molecules15020649

**Published:** 2010-01-28

**Authors:** Sedat Yaşar, Emine Özge Özcan, Nevin Gürbüz, Bekir Çetinkaya, İsmail Özdemir

**Affiliations:** 1 Department of Chemistry, Faculty of Science and Art, Gaziosmanpaşa University, 60240 Tokat, Turkey; E-Mail: syasar44@gmail.com; 2 Department of Chemistry, Faculty of Science and Art, İnönü University, 44280 Malatya, Turkey; E-Mails: eozgeozcan@gmail.com (E.Ö.Ö.); ngurbuz@inonu.edu.tr (N.G.); 3 Department of Chemistry, Faculty of Science, Ege University, 35100 İzmir, Turkey; E-Mail: bekircetinkaya@ege.edu.tr

**Keywords:** carbene, C-C coupling, palladium/tetrahydropyrimidinium, Heck coupling

## Abstract

An efficient and stereoselective catalytic system for the Heck cross coupling reaction using novel 1,3-dialkyl-3,4,5,6-tetrahydropyrimidinium salts (**1**, **LHX**) and Pd(OAc)_2_ loading has been reported. The palladium complexes derived from the salts **1a-f **prepared *in situ* exhibit good catalytic activity in the Heck coupling reaction of aryl bromides under mild conditions.

## 1. Introduction

The Heck reaction, one of the most utilized cross-coupling reactions, is the palladium-catalyzed arylation of an olefin with an aryl halide under basic conditions. Since its independent discovery in the early 1970s by Heck [1] and Mizoroki [2], the Heck reaction has been widely used as a tool for organic synthesis because of its importance in the direct attachment of olefinic groups to aromatic rings [3,4,5]. Numerous review articles on various aspects of the Heck and other cross-coupling reactions with palladium catalysts have been published [6,7,8,9]. Many types of ligands have been explored for the palladium catalysts in the Heck reaction, e.g., phosphine [10,11,12], carbene [13], amine [14] and thiolate [15]. The use of immobilized [16] and non-immobilized [17] ligand free palladium salts for the Mizoroki–Heck reaction with chloro- and bromoarenes is also discussed in the literature. Recently, Gibson *et al.* [18] as well as Jones *et al.* [20] showed that palladium complexes with chiral ligands are well suited as catalysts for the asymmetric Heck reaction. 

Heck coupling reactions often require high temperatures (normally 110–180 °C) to proceed, even with activated aryl bromides [20,21,22,23,24]. Only a few catalyst systems can catalyze Heck–Mizoroki reactions at a temperature below 100 °C [25,26]. The high thermal stability of transition metal complexes of *N*-heterocyclic carbenes (NHCs) makes these complexes particularly suitable for Heck coupling reactions [27,28,29,30,31,32]. The first application of Pd–NHCcomplexes for the Heck reaction of aryl bromides and activated aryl chlorides was reported by Herrmann *et al. *in 1995 [13]. Since then, a number of monodentate and bidentate NHC ligands have been shown to have good activity in Pd-catalyzed Heck reactions [33,34,35,36]. Due to the high stability of metal complexes of NHCs towards heat, oxygen and moisture [37], they have long been the subject of catalytic studies. In addition to the palladium catalyzed Mizoroki–Heck reaction, they have been used for the catalysis of other C–C coupling reactions [38], hydroformylations [39], polymerization reactions [40], olefin metathesis [41], and CH-activation [42]. 

The nature of the NHC ligand has a tremendous influence on the rate of catalyzed reactions. Whilst modifications to the five membered ring of the ligand aryl substituent have been described, relatively little attention has been paid to effect of the ring size. Due to their six-membered ring geometry, tetrahydropyrimidine-2-ylidenes are stronger donating ligands in comparison to their five-membered relatives [43]. The use of tetrahydropyrimidinium ligands in catalytic transformations is limited [44,45,46,47,48]. 

**Scheme 1:**
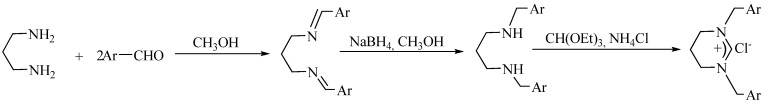
Preparation of tetrahydropyrimidinium salts.

We have previously reported the use of an *in situ* formed imidazolidine, tetrahydropyrimidine, tetrahydrodiazepine, benzimidazole-2-ylidene-palladium(II) system which exhibits high activity in various coupling reactions of aryl bromides and aryl chlorides [49,50,51,52]. In order to find more efficient palladium catalysts we have prepared a series of new 1,3-dialkyl-3,4,5,6- tetrahydropyrimidinium **1a-f** (Scheme 1), and we now report the use of the *in situ* generated catalytic system composed of Pd(OAc)_2_ as palladium source, **1a-f** as carbene precursors and K_2_CO_3_ as a base for cross coupling of aryl bromides with phenylstyrene in DMF/water at 80 °C for 4 h.

## 2. Results and Discussion

### 2.1. Synthesis of tetrahydropyrimidinium salts

A series of symmetrical 1,3-dialkyl-3,4,5,6-tetrahydropyrimidinium salts (**1 = LHX**) were prepared according to known methods [28] as conventional NHC precursors. The symmetrical NHC precursors were prepared according to general reaction pathway depicted in Scheme 1. Treatment of 1,3‑propylenediamine with 2 equivalents of aromatic aldehyde in methanol at room temperature led to the formation of the corresponding diimines. Their reduction with sodium borohydride in methanol, followed by treatment with triethylorthoformate in the presence of ammonium chloride with continuous elimination of ethanol led to the formation of the expected tetrahydropyrimidinium chlorides in excellent yields. 

**Figure 1:**
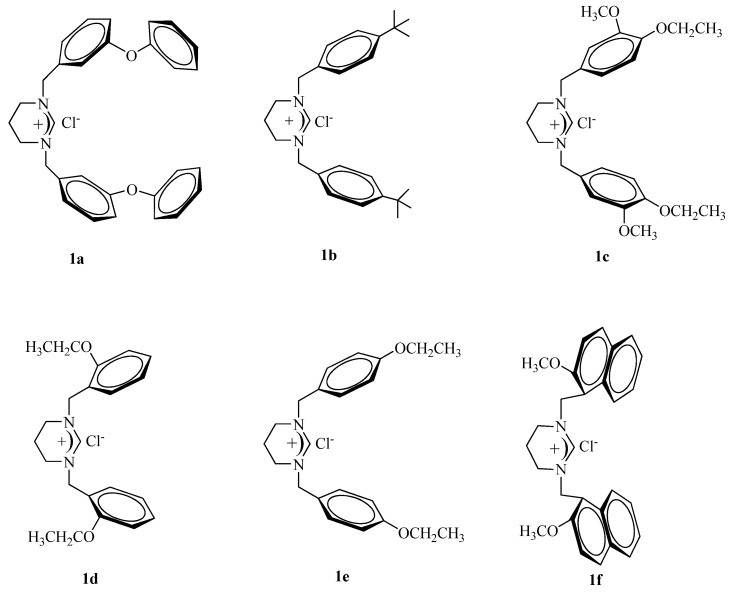
Tetrahydropyrimidinium salts.

The tetrahydropyrimidinium salts (Figure 1) were isolated as colourless solids in very good yields and fully characterized by ^1^H- and ^13^C-NMR, and IR spectroscopies, elemental analyses, and their melting points were determined (see Experimental section). 

The salts are air- and moisture stable both in the solid state and in solution. The NMR values are similar to those found for other tetrahydropyrimidinium salts [45]. Thus, the ^1^H-NMR spectra support the assigned structures; the resonances for the acidic C(2)-H in **1a-f ** were observed as sharp singlets at 10.28, 10.20, 10.08, 9.40, 10.45 and 8.80 ppm, respectively. The position of the ethoxy groups on the phenyl rings also has a strong influence on the acidity of the proton as a chemical shift of 9.40 ppm is observed in the case of the 2-substitution in **1d**, whereas the 4-substitution pattern in **1e** leads to a signal at 10.45 ppm. ^13^C-NMR chemical shifts were also consistent with the proposed structures; the imino carbons of the tetrahydropyrimidinium salts **1a-f** appeared as a typical singlet in the ^1^H-decoupled mode at 158.2, 154.5, 154.1, 157.4, 159.4 and 155.8 ppm, respectively. The IR data for **1a-f **clearly indicate the presence of the –C=N- group with ν(C=N) vibrations at 1,695, 1,701, 1,695, 1,688 and 1,678 cm^-1^, respectively. 

### 2.2. The Heck C-C coupling reaction

The Heck reaction has been shown to be very useful for the preparation of disubstituted olefins in particular. The rate of the coupling is dependent on a variety of parameters such as temperature, solvent, base and catalyst loading. Generally, Heck reactions conducted with tertiary phosphine or NHC complexes required high temperatures (higher than 120 °C) and polar solvents [20,21,22,23,24]. For the choice of base, we surveyed Cs_2_CO_3_, K_2_CO_3_, *t-*BuOK, and K_3_PO_4_. To find optimum conditions a series of experiments was performed with 4-bromoacetophenone and styrene as model compounds. Finally, we found that use of 1% mol Pd(OAc)_2_, 2% mol **1**, 2 equiv K_2_CO_3_ in DMF/H_2_O (1:1) at 80 °C led to the best conversion within 4 h. Since the salts **1a-f** are thermally stable and inert toward air and moisture in the solid state, these properties allowed for catalytic experiments under aerobic conditions and the reactions were performed in air and without degassing the water and DMF prior to use. 

Control experiment indicated that the coupling reaction did not occur in the absence of the salt **1. **Under the determined reaction conditions, a wide range of aryl bromides bearing electron-donating or electron-withdrawing groups react with styrene affording the coupled products in optimum yields. As expected, the use of electron-deficient bromides was beneficial for the conversions. Under those conditions *p*-bromoacetophenone, *p*-bromobenzaldehyde, bromotoluene and *p*-bromoanisole react very cleanly and in goods yields with styrene (Table 1, entries 4, 10, 15 and 22). Enhancement in activity, although less significant, is further observed employing 4-bromo-benzaldehyde instead of 4-bromoacetophenone (Table 1, entries 1–6, 19–24). The substituent effect in the tetrahydropyrimidinium salts indicated that a ethoxy group of the aromatic ring on the *N* atoms increased the yield of product. The catalytic activity of the salts used falls in the order **b** ≈ **d** > **c** > **a** > **e** > **f**. Table 1 summarizes our results from the screening of new tetrahydropyrimidinium salts for the Heck coupling reaction. The procedure is simple and does not require an induction period. This catalytic system provides good conditions for the coupling of aryl bromides under air. It should be noted that in all cases only the *trans *products were selectively obtained as confirmed by ^1^H-NMR. 

**Table 1:** The Heck coupling reaction of aryl bromides with styrene. 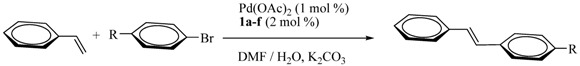

Entry	R	LHX	Yield^a, b, c,d ^(%)
**1**	COCH_3_	**1a**	90
**2**	COCH_3_	**1b**	99
**3**	COCH_3_	**1c**	94
**4**	COCH_3_	**1d**	99
**5**	COCH_3_	**1e**	93
**6**	COCH_3_	**1f**	86
**7**	CH_3_	**1a**	65
**8**	CH_3_	**1b**	82
**9**	CH_3_	**1c**	78
**10**	CH_3_	**1d**	95
**11**	CH_3_	**1e**	70
**12**	CH_3_	**1f**	82
**13**	OCH_3_	**1a**	70
**14**	OCH_3_	**1b**	96
**15**	OCH_3_	**1c**	98
**16**	OCH_3_	**1d**	99
**17**	OCH_3_	**1e**	98
**18**	OCH_3_	**1f**	74
**19**	CHO	**1a**	97
**20**	CHO	**1b**	98
**21**	CHO	**1c**	97
**22**	CHO	**1d**	99
**23**	CHO	**1e**	96
**24**	CHO	**1f**	86
**25**	H	**1a**	62
**26**	H	**1b**	60
**27**	H	**1c**	66
**28**	H	**1d**	85
**29**	H	**1e**	78
**30**	H	**1f**	82

^a ^*Reaction conditions*: 1.0 mmol of R-C_6_H_4_Br-*p*, 1.5 mmol of styrene, 2 mmol K_2_CO_3_, 1 mmol % Pd(OAc)_2_, 2 mmol % **1a-f**, H_2_O (3 mL)-DMF (3 mL); ^b^ Purity of compounds is checked by NMR and yields are based on aryl halide; ^c ^All reactions were monitored by TLC and GC; ^d^ Temperature 80 °C, 4 h.

## 3. Experimental Section

### 3.1. General

All reactions for the preparation of **1 **were carried out under Ar in flame-dried glassware using standard Schlenk-type flasks. The solvents used were purified by distillation over the indicated drying agents and were transferred under Ar: Et_2_O (Na/K alloy), C_2_H_5_OH (Mg). Flash chromatography: Merck silica gel 60 (230-400 mesh). All reagents were purchased from Aldrich Chemical Co. Melting point were determined in glass capillaries under air with an Electrothermal-9200 melting point apparatus. FT-IR spectra were recorded as KBr pellets in the range 400-4,000 cm^-1^ on a ATI UNICAM 1000 spectrometer. ^1^H-NMR and ^13^C-NMR spectra were recorded in CDCl_3 _with tetramethylsilane as an internal reference using a Varian As 400 Merkur spectrometer operating at 400 MHz (^1^H), 100 MHz (^13^C). The NMR studies were carried out in high-quality 5 mm NMR tubes. Signals are quoted in parts per million as δ downfield from tetramethylsilane (δ 0.00) as an internal standard. Coupling constants (*J* values) are given in hertz. NMR multiplicities are abbreviated as follows: s = singlet, d = doublet, t = triplet, m = multiplet signal. All reactions were monitored on an Agilent 6890N GC system by GC-FID with a HP-5 column of 30 m length, 0.32 mm diameter and 0.25 μm film thickness. Column chromatography was performed using silica gel 60 (70-230 mesh). Solvent ratios are given as v/v. Elemental analyses were performed by Turkish Research Council Microlab (Ankara, Turkey).

### 3.2. General preparation of symmetrical 1,3-dialkyl-3,4,5,6-tetrahydropyrimidinium salts

The aromatic aldehyde (20 mmol) and 1,3-propylendiamine (10 mmol) were stirred overnight in methanol (25 mL). The diimine was collected as a white solid, filtered and recrystallized from an alcohol/ether mixture. The diimine (10 mmol) was subsequently reduced by NaBH_4_ (30 mmol) in CH_3_OH (30 mL). The solution was then treated with 1 N HCl, and the organic phase was extracted with CH_2_Cl_2 _(3 x 30 mL). After drying over MgSO_4_ and evaporation, the diamine was isolated as a solid and then treated in a large excess of triethyl orthoformate (50 mL) in the presence of NH_4_Cl (10 mmol) at 110 °C in a distillation apparatus until the removal of ethanol ceased. Upon cooling to RT a colourless solid precipitated, which was collected by filtration and dried under vacuum. The crude product was recrystallized from absolute ethanol to give colourless needles and the solid was washed with diethyl ether (2 × 10 mL) and dried under vacuum. 

*1,3-Bis-(3-phenoxybenzyl)-3,4,5,6-tetrahydropyrimidinium chloride *(**1a**). Yield: 87%, m.p.: 125–126 °C; υ_(CN)_ = 1,695 cm ^-1^; ^1^H-NMR δ: 1.19 (quint, *J = * 4 Hz, 2H, NCH_2_C*H_2_*CH_2_N), 3.23 (t, *J *= 4 Hz, 4H, NC*H_2_*CH_2_C*H_2_*N), 4.89 (s, 4H, C*H_2_*C_6_H_4_OC_6_H_5_), 6.89-7.33 (m, 18 H, CH_2_C_6_*H_4_*OC_6_*H_5_*), 10.28 (s, 1H, NC*H*N); ^13^C{H}NMR δ: 19.2 (NCH_2_*C*H_2_CH_2_N), 42.2 (N*C*H_2_CH_2_*C*H_2_N), 58.4 (*C*H_2_C_6_H_4_OC_6_H_5_), 118.9, 119.4, 123.4, 124.0, 130.1, 131.0, 135.4, 155.2 and 156.7 (CH_2_*C_6_*H_4_O*C_6_*H_5_), 158.2 (N*C*HN); Anal. Calcd. for C_30_H_29_N_2_O_2_Cl: C,74.29; H, 6.03; N, 5.78%; found: C, 74.32; H, 6.08; N, 5.83%.

*1,3-Bis-(4-t-butylbenzyl)-3,4,5,6-tetrahydropyrimidinium chloride* (**1b**). Yield: 85%, m.p.: 315 °C; υ_(CN)_ = 1,701 cm ^-1^; ^1^H-NMR δ: 1.22 (s, 18H, CH_2_C_6_H_4_C(C*H_3_*)_3_-4), 1.90 (quint, *J = * 4 Hz, 2H, NCH_2_C*H_2_*CH_2_N), 3.16 (t, *J *= 4 Hz, 4H, NC*H_2_*CH_2_C*H_2_*N), 4.81 (s, 4H, C*H*_2_C_6_H_4_C(CH_3_)_3_-4), 7.28 (m, 8H, CH_2_C_6_*H_4_*C(CH_3_)_3_-4), 10.20 (s, 1H, NC*H*N); ^13^C{H}NMR δ: 19.2 (NCH_2_*C*H_2_CH_2_N), 31.5 (CH_2_C_6_H_4_C(*C*H_3_)_3_-4), 34.8 (CH_2_C_6_H_4_*C*(CH_3_)_3_-4), 42.1 (N*C*H_2_CH_2_*C*H_2_N), 58.4 (*C*H_2_C_6_H_4_C(CH_3_)_3_-4), 126.2, 128.7, 130.4 and 152.1 (CH_2_*C*_6_H_4_C(CH_3_)_3_-4), 154.5 (N*C*HN); Anal. Calcd. for C_26_H_37_N_2_Cl: C,75.61; H, 9.03; N, 6.78%; found: C, 75.66; H, 9.08; N, 6.83%.

*1,3-Bis-(3-methoxy-4-ethoxybenzyl)-3,4,5,6-tetrahydropyrimidinium chloride *(**1c**). Yield: 89%, m.p.: 177–178 °C; υ_(CN)_ = 1,695 cm^-1^; ^1^H-NMR δ: 1.35 (t, *J *= 4.8 Hz, 6H,CH_2_C_6_H_3_OCH_2_C*H*_3_-4), 1.85 (quint, *J = * 5.6 Hz, 2H, NCH_2_C*H_2_*CH_2_N), 3.14 (t, *J *= 5.6 Hz, 4H, NC*H_2_*CH_2_C*H_2_*N), 3.81 (s, 6H, CH_2_C_6_H_3_OC*H*_3_-3), 3.98 (quart., *J * = 4.8 Hz, 4H, CH_2_C_6_H_3_OC*H*_2_CH_3_-4), 4.71 (s, 4H, C*H*_2_C_6_H_3_(OCH_3_-3)-(OCH_2_CH_3_-4)), 6.70-7.05 (m, 6H, CH_2_C_6_*H*_3_(OCH_3_-3)-(OCH_2_CH_3_-4)), 10.08 (s, 1H, NC*H*N); ^13^C{H}NMR δ: 14.9 (CH_2_C_6_H_3_OCH_2_*C*H_3_-4), 19.2 (NCH_2_*C*H_2_CH_2_N), 41.9 (N*C*H_2_CH_2_*C*H_2_N), 58.5 (*C*H_2_C_6_H_3_(OCH_3_-3)-(OCH_2_CH_3_-4)), 56.7 (CH_2_C_6_H_3_O*C*H_2_CH_3_-4), 64.5 (CH_2_C_6_H_3_O*C*H_3_-3), 121.6, 125.9, 149.0 and 150.0 (CH_2_*C*_6_H_3_(OCH_3_-3)-(OCH_2_CH_3_-4)), 154.1 (N*C*HN); Anal. Calcd. for C_24_H_34_N_2_O_4_Cl: C,64.20; H, 7.41; N, 6.24%; found: C, 64.24; H,7.44; N, 6.27%.

*1,3-Bis-(2-ethoxybenzyl)-3,4,5,6-tetrahydropyrimidinium chloride *(**1d**). Yield: 80 %, m.p.: 171–172 °C; υ_(CN)_ = 1,677 cm^-1^; ^1^H-NMR δ: 1.32 (t, *J *= 8 Hz, 6H,CH_2_C_6_H_4_OCH_2_C*H*_3_-2), 1.94 (quint, *J =* 4 Hz, 2H, NCH_2_C*H_2_*CH_2_N), 3.27 (t, *J *= 4 Hz, 4H, NC*H_2_*CH_2_C*H_2_*N), 3.98 (quart., *J *= 8 Hz, 4H, CH_2_C_6_H_4_OC*H*_2_CH_3_-2), 4.79 (s, 4H, C*H*_2_C_6_H_4_OCH_2_CH_3_-2), 6.81-7.42 (m, 8H, CH_2_C_6_*H*_4_OCH_2_CH_3_-2), 9.40 (s, 1H, NC*H*N); ^13^C{H}NMR δ: 15.1 (CH_2_C_6_H_4_OCH_2_*C*H_3_-2), 19.4 (NCH_2_*C*H_2_CH_2_N), 42.7 (N*C*H_2_CH_2_*C*H_2_N), 54.3 (*C*H_2_C_6_H_4_OCH_2_CH_3_-2), 64.0 (CH_2_C_6_H_4_O*C*H_2_CH_3_-2), 121.2, 121.8, 130.8, 131.6 and 154.7 (CH_2_*C*_6_H_4_OCH_2_CH_3_-2), 157.4 (N*C*HN); Anal. Calcd. for C_22_H_29_N_2_O_2_Cl: C, 62.94; H, 7.52; N, 7.20%; found: C, 62.88; H, 7.54; N, 7.25%.

*1,3-Bis-(4-ethoxybenzyl)-3,4,5,6-tetrahydropyrimidinium chloride* (**1e**). Yield: 85%, m.p.: 199–200 °C; υ_(CN)_ = 1,688 cm^-1^; ^1^H-NMR δ: 1.34 (t, *J *= 6.9 Hz, 6H,CH_2_C_6_H_4_OCH_2_C*H*_3_-4), 1.88 (quint, *J =* 6 Hz, 2H, NCH_2_C*H_2_*CH_2_N), 3.14 (t, *J *= 5.7 Hz, 4H, NC*H_2_*CH_2_C*H_2_*N), 3.36 (quart., *J *= 6.9 Hz, 4H, CH_2_C_6_H_4_OC*H*_2_CH_3_-4), 4.75 (s, 4H, C*H*_2_C_6_H_4_OCH_2_CH_3_-4), 6.77 and 7.31 (d, *J =* 6 Hz, 8H, CH_2_C_6_*H*_4_OCH_2_CH_3_-4), 10.45 (s, 1H, NC*H*N); ^13^C{H}NMR δ: 14.7 (CH_2_C_6_H_4_OCH_2_*C*H_3_-4), 18.9 (NCH_2_*C*H_2_CH_2_N), 41.6 (N*C*H_2_CH_2_*C*H_2_N), 57.8 (*C*H_2_C_6_H_4_OCH_2_CH_3_-4), 63.5 (CH_2_C_6_H_4_O*C*H_2_CH_3_-4), 114.9, 124.9 130.3 and 153.8 (CH_2_*C*_6_H_4_OCH_2_CH_3_-4), 159.4 (N*C*HN); Anal. Calcd. for C_22_H_29_N_2_O_2_Cl: C,62.94; H, 7.52; N, 7.20%; found: C, 62.89; H, 7.53; N, 7.23%.

*1,3-Bis-(2-methoxynaphtomethyl)-3,4,5,6-tetrahydropyrimidinium chloride* (**1f**). Yield: 85%, m.p.: 96–97 °C; υ_(CN)_ = 1,678 cm^-1^. ^1^H-NMR δ: 1.88 (quint, *J =* 6 Hz, 2H, NCH_2_C*H_2_*CH_2_N), 3.24 (t, *J *= 5.7 Hz, 4H, NC*H_2_*CH_2_C*H_2_*N), 3.70 (s, 6H, CH_2_C_10_H_6_OC*H*_3_-2), 5.08 (s, 4H, C*H*_2_C_10_H_6_OCH_3_-2), 7.09-7.94 (m, 12H, CH_2_C_10_*H*_6_OCH_3_-2), 8.80 (s, 1H, NC*H*N); ^13^C{H}NMR δ: 18.0 (NCH_2_*C*H_2_CH_2_N), 42.3 (N*C*H_2_CH_2_*C*H_2_N), 48.8 (*C*H_2_C_10_H_6_OCH_3_-2), 55.8 (CH_2_C_10_H_6_O*C*H_3_-2), 111.4, 112.0, 121.5, 123.6, 127.7, 128.4, 128.5, 131.5, 132.2 and 151.5 (CH_2_*C*_10_H_6_OCH_3_-2), 155.8 (N*C*HN); Anal. Calcd. for C_28_H_29_N_2_O_2_Cl: C,72.95; H, 6.34; N, 6.08%; found: C, 72.99; H, 6.38; N, 6.11%.

### 3.3. General procedure for the Heck Coupling reaction

Pd(OAc)_2 _(1.0 mmol %), 1,3-dialkyl-3,4,5,6-tetrahydropyrimidinium salt** 1** (2 mmol %), aryl bromide (1.0 mmol), styrene (1.5 mmol), K_2_CO_3_ (2 mmol) water (3 mL)-DMF (3 mL) were added to a small Schlenk tube and the mixture was heated at 80 °C for 4 h. At the conclusion of the reaction, the mixture was cooled, extracted with ethyl acetate/hexane (1:5), filtered through a pad of silicagel with copious washing, concentrated and purified by flash chromatography on silica gel. Purity of the compounds was checked by NMR and GC; yields are based on aryl bromide.

## 4. Conclusions

In summary, we have synthesized new 3,4,5,6‑tetrahydropyrimidinium salts as precursors of *N*-heterocyclic carbenes upon deprotonation. They have been associated with Pd(OAc)_2_ to generate catalytic species. Such Pd(OAc)_2_- tetrahydropyrimidinium salts system could successfully catalyze the Heck reactions available for an array of substrates including a wide range of aryl bromides bearing electron-donating or electron-withdrawing groups with high activity and stability in aerial atmosphere. Detailed investigations focusing on NHC (imidazolidine, tetrahydropyrimidine, benzimidazolidine) and catalytic activity in this and other coupling reactions are ongoing. 
